# Childhood adiposity and novel subtypes of adult-onset diabetes: a Mendelian randomisation and genome-wide genetic correlation study

**DOI:** 10.1007/s00125-023-05883-x

**Published:** 2023-02-27

**Authors:** Yuxia Wei, Tom G. Richardson, Yiqiang Zhan, Sofia Carlsson

**Affiliations:** 1 Institute of Environmental Medicine, Karolinska Institutet, Stockholm, Sweden; 2 MRC Integrative Epidemiology Unit (IEU), Population Health Sciences, Bristol Medical School, University of Bristol, Bristol, UK; 3 School of Public Health (Shenzhen), Sun Yat-Sen University, Shenzhen, China

**Keywords:** Epidemiology, Obesity, Prediction, Prevention, Type 2 diabetes, Weight regulation

## Abstract

**Aims/hypothesis:**

We investigated whether the impacts of childhood adiposity on adult-onset diabetes differ across proposed diabetes subtypes using a Mendelian randomisation (MR) design.

**Methods:**

We performed MR analysis using data from European genome-wide association studies of childhood adiposity, latent autoimmune diabetes in adults (LADA, proxy for severe autoimmune diabetes), severe insulin-deficient diabetes (SIDD), severe insulin-resistant diabetes (SIRD), mild obesity-related diabetes (MOD) and mild age-related diabetes (MARD).

**Results:**

Higher levels of childhood adiposity had positive genetically predicted effects on LADA (OR 1.62, 95% CI 1.05, 2.52), SIDD (OR 2.11, 95% CI 1.18, 3.80), SIRD (OR 2.76, 95% CI 1.60, 4.75) and MOD (OR 7.30, 95% CI 4.17, 12.78), but not MARD (OR 1.06, 95% CI 0.70, 1.60).

**Conclusions/interpretation:**

Childhood adiposity is a risk factor not only for adult-onset diabetes primarily characterised by obesity or insulin resistance, but also for subtypes primarily characterised by insulin deficiency or autoimmunity. These findings emphasise the importance of preventing childhood obesity.

**Graphical abstract:**

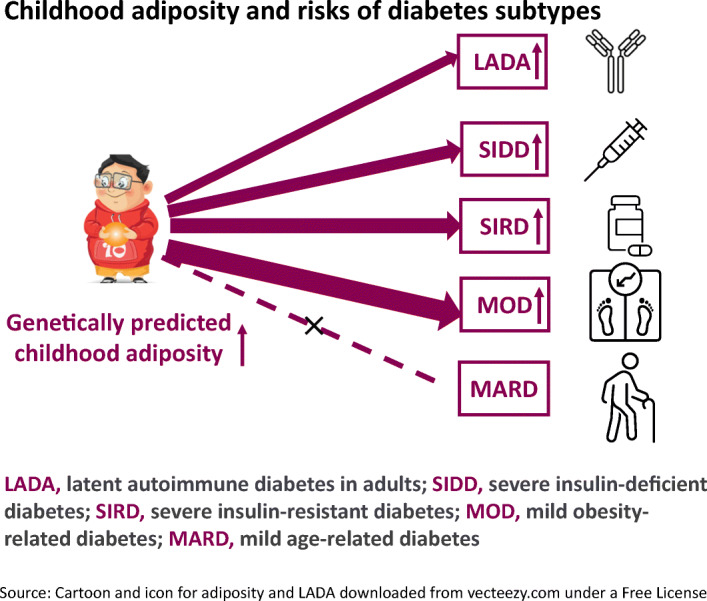

**Supplementary Information:**

The online version contains peer-reviewed but unedited supplementary material available at 10.1007/s00125-023-05883-x.

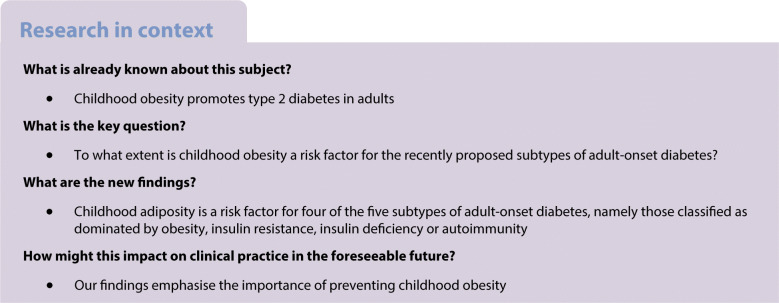



## Introduction

In 2018, a ground-breaking study identified five novel subtypes of adult-onset diabetes: severe autoimmune diabetes (SAID, including type 1 diabetes and latent autoimmune diabetes in adults [LADA]) and four subtypes of type 2 diabetes (severe insulin-deficient diabetes [SIDD], severe insulin-resistant diabetes [SIRD], mild obesity-related diabetes [MOD] and mild age-related diabetes [MARD]) [1]. These subtypes differ in their clinical characteristics, complications and genetic backgrounds [1, 2]. It is unclear if they also differ in modifiable risk factors.

The prevalence of childhood obesity is rising worldwide. Childhood adiposity has been linked to several chronic diseases including type 1 diabetes in children and type 2 diabetes [3, 4]; however, it has never been investigated in relation to the recently proposed subtypes of adult-onset diabetes. We aimed to compare the genetically predicted effects of childhood body size on different diabetes subtypes using a Mendelian randomisation (MR) design and to explore the shared genetics between adiposity and these subtypes.

## Methods

We integrated a two-sample MR design and genome-wide genetic correlation analyses using summary statistics from genome-wide association studies (GWAS) of adiposity and diabetes subtypes in adults. An MR study uses independent SNPs as instrumental variables (IVs) for causal inference. IVs should be associated with the exposure, but not associated with the outcome directly or through confounders [5]. No ethical approval was required as we used GWAS summary statistics.

### GWAS of adiposity

**Fig. 1:**
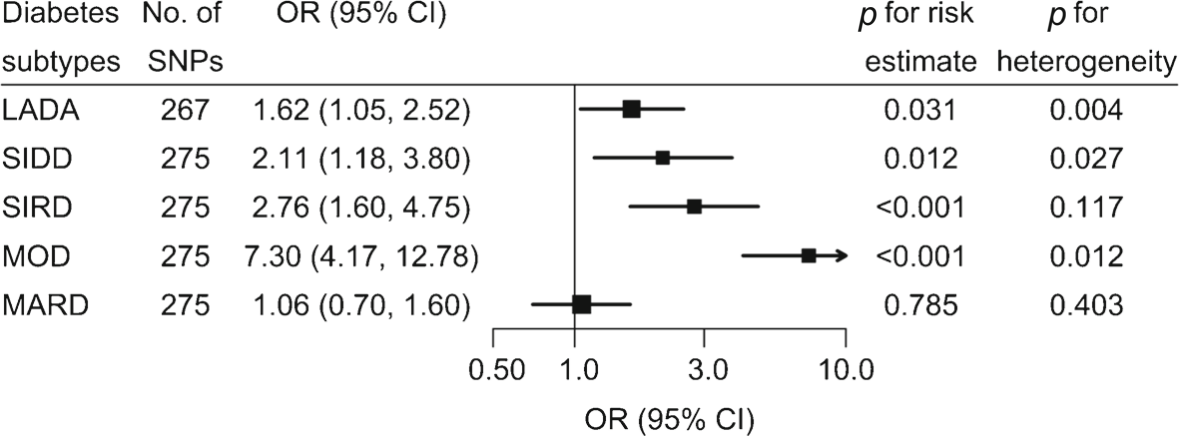
MR analyses of diabetes subtypes in adults in relation to each change in category (thinner, about average or plumper) of childhood body size. The analyses were based on the IVW method

We extracted summary statistics for childhood body size from a GWAS of 453,169 European participants who self-reported body size (thinner, about average, and plumper) at the age of 10 years in the UK Biobank study [3]. The GWAS analysis was adjusted for sex, age at baseline, type of genotyping array and month of birth and identified 295 independent SNPs (linkage disequilibrium [LD] threshold: *r*^2^<0.001 [3]) for childhood body size. Among them, 267 SNPs (including 12 proxy SNPs in LD of *r*^2^≥0.08 with the unavailable SNPs) and 275 SNPs (including 17 proxy SNPs) were available in the GWAS of LADA and other diabetes subtypes (described below), respectively, and were used as IVs in the MR analysis (electronic supplementary material [ESM] Fig. 1, ESM Tables 1 and 2). These IVs have been validated to be robust in reflecting measured childhood adiposity [3]. Summary statistics for adult BMI were obtained from the GWAS of 359,983 European individuals in UK Biobank (https://broad-ukb-sumstats-us-east-1.s3.amazonaws.com/round2/additive-tsvs/21001_irnt.gwas.imputed_v3.both_sexes.tsv.bgz, accessed 1 Aug 2022).

### GWAS of diabetes subtypes

The only GWAS of novel diabetes subtypes was conducted in individuals with newly diagnosed diabetes and individuals without diabetes in Sweden, with sample sizes of 3196, 3937, 3874, 4118 and 5605 for the analysis of SAID, SIDD, SIRD, MOD and MARD, respectively [2]. The subtypes were classified based on autoimmunity, age, BMI, HbA_1c_, beta cell function and insulin resistance at diabetes diagnosis [1]. One GWAS of LADA has been performed, in 8581 European individuals [6]. To increase statistical power, we used summary statistics from the LADA GWAS rather than the GWAS of SAID (80% of SAID patients have LADA) for subsequent analyses. Therefore, the analysed subtypes included LADA, SIDD, SIRD, MOD and MARD. The GWAS analyses were adjusted for sex and principal components [2, 6].

### Statistical analysis

We performed MR analyses to assess the association of genetically predicted childhood body size with different diabetes subtypes. The inverse-variance weighted (IVW) [5] method was the main estimator, supplemented by other MR estimators including robust IVW [7], weighted median [8], MR-Egger [9] and Mendelian randomisation pleiotropy residual sum and outlier (MR-PRESSO) [10]. We also performed leave-one-out analyses by excluding one SNP from the IVs each time to test the robustness of the MR results.

We calculated the overall genetic correlation (*r*_*g*_) between childhood body size and diabetes subtypes, which incorporates genome-wide contribution of genetic variation, using linkage disequilibrium score regression (LDSC; see ESM Methods), using full sets of GWAS summary statistics (except for HLA regions). For comparison reasons, we also calculated the *r*_*g*_ between adult BMI and diabetes.

MR analyses were performed using *MendelianRandomization* and *MR-PRESSO* package in R 4.0.4 (https://www.R-project.org/), while *r*_*g*_ was calculated using LDSC v1.0.1 [11, 12].

## Results

In the MR analyses, genetically predicted body size during childhood had a positive genetically predicted effect on all diabetes subtypes except MARD. The magnitude of the increased risks varied, with ORs of 1.62 (95% CI 1.05, 2.52) for LADA, 2.11 (95% CI 1.18, 3.80) for SIDD, 2.76 (95% CI 1.60, 4.75) for SIRD and 7.30 for MOD (95% CI 4.17, 12.78; Fig. 1, ESM Fig. 2). Such increased risks were consistent across different MR estimators (ESM Fig. 3). MR-Egger detected no horizontal pleiotropy (*p*>0.05), indicating no violation of the MR assumption. MR-PRESSO detected rs10498713 and rs1000471 as the outliers for the analysis of SIDD and MOD, respectively, but removing the outliers changed the estimates only slightly (ESM Fig. 3). There was no major change in the risk estimates by leaving one SNP out each time from the MR analysis, although the 95% CI of the OR for LADA crossed 1 when rs1421085 (nearest gene: *FTO*) or rs663129 (nearest gene: *MC4R*) was excluded from the IVs (ESM Fig. 4).

**Fig. 2:**
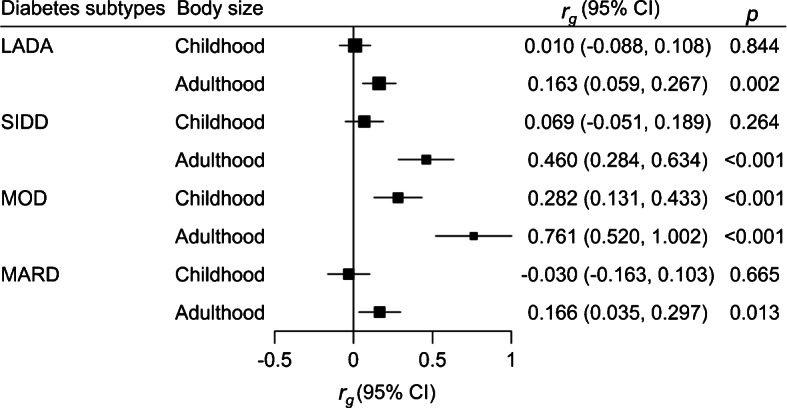
Genetic correlation between body size over the life course and different subtypes of diabetes in adults. The *r*_*g*_ for SIRD was not estimated because there was no genome-wide significant SNP for this subtype

Childhood body size was genetically correlated with MOD (*r*_*g*_: 0.282, *p*<0.001) but not with other subtypes of diabetes (Fig. 2). In comparison, adult BMI was genetically correlated with all diabetes subtypes, with the highest correlation with MOD (*r*_*g*_: 0.761) and lowest correlation with LADA (*r*_*g*_: 0.163) and MARD (*r*_*g*_: 0.166).

## Discussion

Except for MARD, our MR analyses based on large European GWAS data suggest that a larger childhood body size is a risk factor for adult-onset diabetes. The genetic impact is greatest on MOD, but adverse effects were also observed for subtypes characterised by autoimmunity (LADA) or severe insulin deficiency (SIDD). Except for MOD, there was no genetic overlap between childhood adiposity and adult-onset diabetes.

Previous MR studies have found that genetically predicted childhood body size is a risk factor for both type 1 diabetes (OR 2.05, 95% CI 1.21, 4.42, mean age at diagnosis 16.57 years) [4] and type 2 diabetes (OR 2.32, 95% CI 1.76, 3.05) [3]. We extend these findings by demonstrating that childhood adiposity is a risk factor for four out of the five recently proposed diabetes subtypes. The link between childhood body size and SIRD is expected, given the adverse effects of adiposity on insulin sensitivity [1]. The smaller OR for SIRD than for MOD suggests that non-obesity-related and/or non-genetic effects may be the main factors underlying the development of SIRD. Interestingly, children with higher levels of adiposity also had higher risks of LADA and SIDD, both of which are characterised by insulin deficiency. This phenomenon may be explained by the fact that impaired insulin secretion is affected jointly by ectopic fat in the pancreas and insulin resistance [13].

Previous MR analyses have found that adult obesity is a risk factor for LADA, SIDD, SIRD and MOD [2, 14]. Children with overweight/obesity often become obese adults, and adult obesity is most likely a mediator in the link between childhood obesity and adult-onset diabetes. This study is the first to investigate genetic correlations between childhood/adult adiposity and different diabetes subtypes. The weak genetic correlation between childhood obesity and adult diabetes indicates that the genes promoting childhood adiposity are largely distinct from those promoting diabetes during adulthood. Adult obesity and diabetes, on the other hand, had a moderate genetic correlation, which was consistent with the results of previous twin studies [15], suggesting that part of this link is explained by shared genetic factors. We found that such genetic overlaps were strongest for MOD and weakest for subtypes characterised by insulin deficiency (LADA and SIDD). In this context it should be noted that the MR-Egger estimator did not identify any potential pleiotropy for the association between childhood body size and any diabetes subtype, and the MR-PRESSO and leave-one-out analyses also suggested that our findings are robust. This indicates no violation of the MR assumption of IVs not affecting the outcome through a pathway outside the exposure. These results together support a causal effect of childhood body size on different subtypes of adult-onset diabetes.

The strength of this study is the use of GWAS data for type 2 diabetes subtypes from the same study population and the unified method of GWAS analysis. This increases the comparability across diabetes subtypes and reveals potentially different mechanisms linking childhood adiposity to different subtypes. In addition, the calculation of genetic correlation with childhood/adult adiposity provides deeper insights into the genetic links between life course adiposity and diabetes subtypes. No GWAS of diabetes subtypes among non-European populations have been carried out and it remains uncertain whether our findings can be extrapolated to such populations. In addition, the lack of GWAS of adult-onset type 1 diabetes precludes us from analysing type 1 diabetes. We could not assess direct and indirect effects of childhood adiposity using a multivariable MR framework [3, 4], given that adulthood adiposity was used to define the subtypes. Last, we acknowledge that the concept of LADA is debated and it has been argued that LADA may include a mix of type 1 and type 2 diabetes [16].

In conclusion, our MR analyses indicate that childhood adiposity is a risk factor for four of the five proposed novel subtypes of adult-onset diabetes, regardless of whether they are classified as being primarily characterised by autoimmunity, insulin deficiency, insulin resistance or obesity.

## Supplementary information

ESM (PDF 1.00 kb)

## Data Availability

The full set of summary statistics for childhood body size is available on request. GWAS summary statistics for adult BMI were downloaded from https://broad-ukb-sumstats-us-east-1.s3.amazonaws.com/round2/additive-tsvs/21001_irnt.gwas.imputed_v3.both_sexes.tsv.bgz. GWAS summary statistics for different subtypes of diabetes were downloaded from the GWAS Catalog (https://www.ebi.ac.uk/gwas/).

## Abbreviations

GWAS: Genome-wide association studies
IVs: Instrumental variables
IVW: Inverse-variance weighted
LADA: Latent autoimmune diabetes in adults
LD: Linkage disequilibrium
LDSC: Linkage disequilibrium score regression
MARD: Mild age-related diabetes
MOD: Mild obesity-related diabetes
MR: Mendelian randomisation
MR-PRESSO: Mendelian randomisation pleiotropy residual sum and outlier
SAID: Severe autoimmune diabetes
SIDD: Severe insulin-deficient diabetes
SIRD: Severe insulin-resistant diabetes
