# Supplementary material for: Childhood adiposity and novel subtypes of adult-onset diabetes: a Mendelian randomisation and genome-wide genetic correlation study
Source: Diabetologia. 2023 Feb 27;66(6):1052–6. doi: 10.1007/s00125-023-05883-x (PMC10163070; doi:10.1007/s00125-023-05883-x)
Supplement: Supplementary file 1 — (PDF 1.00 kb) [file 125_2023_5883_MOESM1_ESM.pdf]

ESM Methods. The calculation of overall genetic correlation (r_g) between adiposity and diabetes subtypes

r_g quantifies the shared genetics between two traits, incorporating genome-wide contribution of genetic variation. We calculated r_g between childhood body size/adult BMI and diabetes subtypes using linkage disequilibrium score regression (LDSC, GitHub - bulik/ldsc: LD Score Regression (LDSC))[1], based on the full sets of GWAS (genome-wide association study) summary statistics (except for HLA regions). LD (linkage disequilibrium) scores used in this study were pre-computed from the 1000 Genomes Project reference panel, and no r^2 cut-off was set in the calculation of LD scores[2]. After merging with reference panel LD, a total of 995396, 1069913, 1070334, and 1069820 SNPs with valid alleles were involved in the calculation of r_g between childhood body size and LADA (latent autoimmune diabetes in adults), SIDD (severe insulin-deficient diabetes), MOD (mild obesity-related diabetes), and MARD (mild age-related diabetes), respectively. A total of 991290, 1010813, 1011215, and 1010739 SNPs were used in the calculation of r_g between adult BMI and LADA, SIDD, MOD, and MARD, respectively.

ESM Table 1. Detailed information on 267 instrumental variables for MR analysis on childhood body size and LADA

SNP	Effect allele	Other allele	SNP-childhood body size association			
			BETA	SE	*p*	F statistic
rs41310284	C	A	0.0206	0.0023	1.4E-18	77
rs11256627	G	A	-0.0089	0.0015	9.5E-09	33
rs2939931	T	C	-0.0079	0.0014	2.2E-08	31
rs1061072	G	A	0.0127	0.0023	2.4E-08	31
rs7084503	T	C	0.0129	0.0014	5.2E-20	84
rs4572029	A	G	0.0111	0.0017	2.2E-10	40
rs10823504	G	A	0.0159	0.0029	3.4E-08	30
rs2242258	T	C	-0.0092	0.0016	8.5E-09	33
rs17399739	A	G	-0.0210	0.0028	3.9E-14	57
rs10887571	C	T	-0.0080	0.0014	1.9E-08	32
rs11215403	G	A	0.0132	0.0016	5.5E-16	66
rs7123283	C	T	0.0083	0.0014	3.7E-09	35
rs10790809	A	G	-0.0097	0.0014	7.0E-12	47
rs56133711	G	A	-0.0162	0.0016	3.1E-24	103
rs4267058	T	C	0.0088	0.0014	1.2E-09	37
rs661878	A	G	0.0138	0.0021	2.5E-11	45
rs3181269	C	T	0.0093	0.0016	6.4E-09	34
rs7951870	T	C	-0.0116	0.0019	5.4E-10	39
rs12798028	C	T	-0.0146	0.0014	1.3E-24	105
rs11040333	G	A	-0.0085	0.0015	3.1E-08	31
rs2958542	C	T	0.0082	0.0015	1.8E-08	32
rs2303384 [a]	C	T	0.0127	0.0015	2.9E-18	76
rs10791902	C	T	-0.0079	0.0014	3.6E-08	30
rs10896348	T	C	0.0119	0.0016	3.3E-14	58
rs10796828	T	G	-0.0113	0.0015	6.9E-15	61
rs61936936	A	T	-0.0136	0.0023	6.2E-09	34
rs7305424	A	T	-0.0104	0.0015	2.4E-12	49
rs2187642	A	C	-0.0112	0.0014	1.0E-14	60
rs12308065	A	G	-0.0083	0.0015	1.2E-08	32
rs28629903	T	C	0.0091	0.0014	1.3E-10	41
rs10841379	A	G	-0.0084	0.0015	2.7E-08	31
rs10842356	A	T	0.0082	0.0014	4.1E-09	35
rs61937656	G	A	0.0117	0.0017	2.8E-12	49
rs116293915 [a]	C	A	-0.0080	0.0014	1.5E-08	32
rs7958241	A	G	-0.0131	0.0015	8.6E-19	78
rs7132908	G	A	-0.0313	0.0014	1.6E-104	471
rs836179	A	G	0.0098	0.0015	1.4E-11	46
rs7306710	T	C	0.0100	0.0014	1.6E-12	50
rs10860295	T	C	-0.0085	0.0014	1.6E-09	36
rs55726687	G	A	-0.0145	0.0017	3.2E-17	71
rs1552759	T	C	0.0092	0.0015	6.2E-10	38
rs7989098	T	C	-0.0121	0.0016	1.0E-13	55
rs9652090	G	T	-0.0083	0.0014	4.0E-09	35
rs1933437	G	A	0.0142	0.0014	1.2E-22	96

SNP	Effect allele	Other allele	SNP-childhood body size association			
			BETA	SE	*p*	F statistic
rs9603697	C	T	-0.0130	0.0015	4.6E-18	75
rs9594686	C	T	0.0107	0.0018	6.4E-09	34
rs12429545	G	A	-0.0206	0.0021	1.2E-22	96
rs9538146	A	G	-0.0116	0.0014	2.0E-16	68
rs1333010	G	A	0.0121	0.0014	4.8E-17	70
rs1576655	A	C	-0.0118	0.0015	4.4E-16	66
rs78420139	G	A	0.0170	0.0031	3.5E-08	30
rs3993347 [a]	T	C	0.0086	0.0015	4.4E-09	34
rs7161424	G	A	-0.0102	0.0014	5.2E-13	52
rs1865719	A	G	-0.0109	0.0015	8.5E-14	56
rs10133279	C	T	-0.0090	0.0014	2.8E-10	40
rs7145052	C	T	-0.0083	0.0014	3.7E-09	35
rs7159126	T	C	0.0087	0.0016	2.4E-08	31
rs72755233	G	A	-0.0176	0.0022	2.3E-15	63
rs824207	A	G	-0.0093	0.0014	3.3E-11	44
rs62048187	G	C	-0.0084	0.0015	4.4E-08	30
rs7163692	C	G	0.0083	0.0015	1.5E-08	32
rs8030456	C	T	0.0213	0.0017	2.8E-37	163
rs7162542	C	G	0.0093	0.0014	4.0E-11	44
rs3817428	C	G	-0.0108	0.0016	1.3E-11	46
rs1000471	C	T	-0.0095	0.0017	3.6E-08	30
rs2970356	C	G	-0.0105	0.0016	4.1E-11	44
rs55880046	T	G	0.0299	0.0020	3.9E-50	222
rs4432271	C	T	-0.0175	0.0021	9.0E-17	69
rs9922288	A	G	0.0105	0.0017	3.0E-10	40
rs62037365	C	G	-0.0137	0.0014	1.4E-21	91
rs4889630	T	C	0.0124	0.0018	1.7E-12	50
rs2238435	C	G	-0.0165	0.0014	3.0E-30	131
rs4783789	T	C	0.0096	0.0017	1.2E-08	32
rs1421085	T	C	-0.0474	0.0014	5.7E-242	1103
rs594585	T	G	0.0080	0.0014	3.4E-08	30
rs7672	C	G	0.0090	0.0016	7.9E-09	33
rs4985555	A	G	0.0087	0.0014	4.4E-10	39
rs11642090	T	C	-0.0117	0.0015	1.0E-15	64
rs72819571	G	T	0.0119	0.0015	7.3E-16	65
rs3815156	A	G	-0.0103	0.0018	2.3E-08	31
rs12601380	A	C	0.0078	0.0014	3.9E-08	30
rs9299	C	T	-0.0096	0.0015	7.7E-11	42
rs17637472	G	A	-0.0109	0.0014	4.2E-14	57
rs7217460	G	A	0.0094	0.0017	2.4E-08	31
rs12941038	C	T	-0.0095	0.0017	1.1E-08	33
rs2246623	C	T	0.0089	0.0014	2.3E-10	40
rs67603370	G	A	-0.0158	0.0027	3.7E-09	35
rs11150745	A	G	0.0122	0.0015	7.5E-16	65
rs7503580	C	T	-0.0111	0.0019	8.9E-09	33
rs1808579	C	T	0.0092	0.0014	7.0E-11	43

SNP	Effect allele	Other allele	SNP-childhood body size association			
			BETA	SE	*p*	F statistic
rs7237444	G	A	0.0101	0.0015	3.2E-11	44
rs7239114	G	A	-0.0135	0.0014	1.7E-21	91
rs68015088	G	A	0.0081	0.0015	4.3E-08	30
rs12606230	T	C	-0.0127	0.0017	1.7E-14	59
rs663129	G	A	-0.0333	0.0017	6.8E-90	404
rs8096658	C	G	0.0090	0.0014	2.0E-10	40
rs1013737	G	C	-0.0106	0.0014	3.4E-14	57
rs4545941	T	C	-0.0109	0.0019	1.1E-08	33
rs116399833	C	A	-0.0123	0.0017	5.3E-13	52
rs4808961	C	G	0.0089	0.0015	1.1E-09	37
rs3810304	A	G	0.0103	0.0017	8.8E-10	38
rs1800437	G	C	0.0118	0.0018	2.6E-11	44
rs3810291	G	A	-0.0147	0.0015	6.4E-23	97
rs601338	G	A	0.0094	0.0014	2.1E-11	45
rs3013431	C	T	0.0087	0.0014	1.3E-09	37
rs12132598	A	G	-0.0083	0.0015	2.2E-08	31
rs7536458	T	G	-0.0101	0.0016	1.9E-10	41
rs11205303	T	C	0.0096	0.0014	1.8E-11	45
rs35588936	C	T	0.0181	0.0029	2.9E-10	40
rs12045879	C	T	0.0099	0.0015	5.3E-11	43
rs12748436	C	G	-0.0156	0.0026	3.5E-09	35
rs543874	A	G	-0.0471	0.0017	6.0E-163	740
rs4074404	T	A	-0.0127	0.0019	4.3E-11	43
rs16839832	G	T	-0.0141	0.0026	3.5E-08	30
rs9438393	A	G	0.0103	0.0014	3.4E-13	53
rs212517	T	A	0.0088	0.0014	9.6E-10	37
rs7354849	A	G	-0.0082	0.0014	6.8E-09	34
rs2356864	G	A	-0.0086	0.0014	8.8E-10	38
rs630602	G	C	-0.0101	0.0014	1.8E-12	50
rs12140153	G	T	0.0218	0.0025	6.0E-19	79
rs2767486	A	G	-0.0154	0.0017	1.2E-18	78
rs2229330	T	G	-0.0197	0.0027	2.3E-13	54
rs7522014	A	G	0.0093	0.0015	9.1E-10	38
rs2755253	C	T	0.0087	0.0015	1.5E-08	32
rs2175171	G	C	-0.0078	0.0014	3.6E-08	30
rs11209943	A	G	-0.0183	0.0014	8.8E-38	165
rs12042908	A	G	0.0275	0.0014	2.5E-84	379
rs34517439	C	A	-0.0138	0.0022	2.0E-10	40
rs6577497	A	T	0.0081	0.0014	1.8E-08	32
rs11165687	C	T	-0.0081	0.0014	1.0E-08	33
rs16996644	C	G	-0.0193	0.0021	4.8E-20	84
rs947088	G	T	-0.0106	0.0016	1.0E-11	46
rs8117463	G	A	0.0089	0.0015	2.9E-09	35
rs73085586	G	A	-0.0097	0.0018	3.0E-08	31
rs2281148	T	C	-0.0098	0.0016	1.5E-09	37
rs2207894	C	T	0.0144	0.0018	7.7E-16	65

SNP	Effect allele	Other allele	SNP-childhood body size association			
			BETA	SE	*p*	F statistic
rs200744777 [a]	T	G	-0.0121	0.0014	1.5E-17	73
rs8130408	A	C	-0.0090	0.0016	2.3E-08	31
rs13047416	C	G	0.0123	0.0015	1.8E-17	72
rs78907487	A	C	-0.0124	0.0020	3.9E-10	39
rs9610387	G	A	0.0138	0.0025	3.7E-08	30
rs6001872	A	G	0.0125	0.0015	1.7E-17	72
rs9611560	T	C	0.0093	0.0016	7.6E-09	33
rs1384660	G	A	0.0158	0.0018	1.8E-18	77
rs7424771	G	A	0.0089	0.0014	2.2E-10	40
rs7606059 [a]	T	C	-0.0105	0.0015	2.1E-12	49
rs11891707	T	C	0.0121	0.0021	3.8E-09	35
rs636402 [a]	T	C	0.0083	0.0014	5.9E-09	34
rs3791478	T	C	0.0125	0.0023	3.3E-08	31
rs1476698	A	G	0.0082	0.0014	1.4E-08	32
rs10182458	A	G	-0.0359	0.0014	3.5E-145	658
rs6719507	G	A	0.0078	0.0014	2.9E-08	31
rs62134189	A	G	0.0142	0.0023	1.0E-09	37
rs2902142	C	T	0.0087	0.0014	1.7E-09	36
rs2539692	T	A	0.0081	0.0014	1.8E-08	32
rs1177279	A	G	0.0088	0.0016	1.6E-08	32
rs2141004	A	C	0.0100	0.0016	2.5E-10	40
rs12992672	G	A	-0.0433	0.0019	4.0E-121	548
rs7565437	T	C	0.0082	0.0014	9.5E-09	33
rs112898427 [a]	C	T	0.0103	0.0015	2.5E-11	45
rs2867116	C	A	-0.0123	0.0020	1.4E-09	37
rs12713889	T	C	0.0117	0.0015	3.8E-15	62
rs2594994	T	A	0.0154	0.0018	3.1E-17	71
rs11925138	G	A	0.0132	0.0024	3.1E-08	31
rs7625768	G	A	-0.0102	0.0015	1.5E-11	46
rs1199333	G	T	0.0135	0.0018	5.9E-14	56
rs59714050	T	A	-0.0228	0.0028	4.9E-16	66
rs355748	G	T	-0.0099	0.0014	6.8E-12	47
rs7633995	A	G	-0.0145	0.0025	4.9E-09	34
rs10937241	A	G	-0.0109	0.0019	1.1E-08	33
rs7619139	T	A	-0.0098	0.0014	6.5E-12	47
rs1402989	C	T	-0.0078	0.0014	2.7E-08	31
rs2268762	A	G	-0.0080	0.0014	2.6E-08	31
rs754635	C	G	-0.0159	0.0022	5.6E-13	52
rs2034963	G	C	0.0111	0.0015	6.7E-14	56
rs35926495	C	T	-0.0091	0.0015	4.3E-10	39
rs3774604	C	T	0.0079	0.0015	4.9E-08	30
rs2629881	C	T	-0.0106	0.0017	3.8E-10	39
rs4688359 [a]	C	T	0.0130	0.0014	2.1E-19	81
rs538579	G	C	-0.0084	0.0015	2.4E-08	31
rs4677156	A	T	0.0092	0.0017	3.7E-08	30
rs1666132	C	T	0.0083	0.0014	6.7E-09	34

SNP	Effect allele	Other allele	SNP-childhood body size association			
			BETA	SE	*p*	F statistic
rs1357798	C	T	-0.0094	0.0017	4.1E-08	30
rs6783281	A	G	-0.0096	0.0015	5.5E-10	38
rs7355953	T	C	-0.0152	0.0017	7.4E-19	79
rs2735556	T	C	0.0172	0.0022	5.1E-15	61
rs7377083	C	A	-0.0137	0.0014	5.3E-22	93
rs2968973	C	T	0.0099	0.0015	1.1E-11	46
rs35189091	A	G	0.0090	0.0015	5.7E-10	38
rs11727676	T	C	0.0131	0.0024	3.0E-08	31
rs34811474	G	A	0.0102	0.0017	8.2E-10	38
rs7656673	A	G	-0.0116	0.0014	6.2E-16	65
rs34722008	G	A	0.0083	0.0015	1.8E-08	32
rs7439324	C	T	0.0108	0.0019	1.5E-08	32
rs12641981	C	T	-0.0222	0.0014	1.8E-55	246
rs788858	A	G	0.0123	0.0015	1.7E-15	63
rs77960	G	A	0.0105	0.0015	1.7E-12	50
rs4958568	G	A	0.0100	0.0016	2.1E-10	40
rs7719067	A	G	0.0135	0.0014	1.8E-21	91
rs7711823	A	G	0.0084	0.0015	9.3E-09	33
rs918472	G	A	-0.0099	0.0016	2.4E-10	40
rs3936511	A	G	0.0108	0.0018	1.1E-09	37
rs6449532	C	T	0.0111	0.0015	3.3E-14	58
rs9291816	C	T	0.0134	0.0015	4.2E-19	80
rs39862	T	C	0.0116	0.0016	7.5E-14	56
rs2307111	T	C	0.0087	0.0014	1.1E-09	37
rs1422067	C	T	0.0109	0.0016	3.8E-11	44
rs2115885	G	A	0.0106	0.0017	9.2E-10	37
rs12214497	G	T	0.0105	0.0015	9.4E-13	51
rs34260097	T	G	-0.0178	0.0017	2.5E-26	113
rs7759938	C	T	-0.0100	0.0015	2.9E-11	44
rs7753558	C	A	0.0091	0.0015	5.2E-10	39
rs1452991	G	A	-0.0101	0.0015	5.3E-12	48
rs796915	C	G	-0.0129	0.0015	2.3E-17	72
rs62425398	C	A	-0.0154	0.0023	1.5E-11	46
rs10498713	G	T	-0.0118	0.0020	2.0E-09	36
rs35162296	C	T	-0.0201	0.0023	1.0E-18	78
rs34196306	G	C	-0.0200	0.0023	2.3E-18	76
rs3131336	C	T	-0.0191	0.0022	2.0E-18	77
rs3129942	G	T	-0.0139	0.0016	2.7E-18	76
rs73422097	A	G	-0.0106	0.0015	3.3E-12	49
rs76187039	G	T	-0.0113	0.0021	3.5E-08	30
rs3798519	A	C	-0.0250	0.0018	8.3E-43	188
rs1775255	G	T	-0.0133	0.0014	2.1E-21	90
rs12110721	G	A	-0.0189	0.0019	1.5E-23	100
rs9370527	G	A	-0.0095	0.0016	6.4E-09	34
rs435775	A	G	-0.0101	0.0016	4.9E-10	39
rs6931604	C	T	-0.0083	0.0014	6.2E-09	34

SNP	Effect allele	Other allele	SNP-childhood body size association			
			BETA	SE	*p*	F statistic
rs6974282	C	T	0.0101	0.0018	1.6E-08	32
rs7808296	C	T	-0.0100	0.0015	2.8E-11	44
rs262338	G	T	-0.0088	0.0014	4.7E-10	39
rs10953577	T	C	-0.0086	0.0014	3.0E-09	35
rs67679818	C	T	0.0078	0.0014	4.9E-08	30
rs6979832	A	G	-0.0094	0.0014	2.3E-11	45
rs11525873	T	C	0.0175	0.0024	1.3E-13	55
rs2722406	C	T	-0.0122	0.0016	4.4E-15	61
rs4723263	G	C	-0.0083	0.0014	3.7E-09	35
rs10234366	G	A	-0.0140	0.0023	1.1E-09	37
rs1852006	G	A	0.0088	0.0015	1.9E-09	36
rs75001243 [a]	C	T	-0.0137	0.0015	5.1E-21	89
rs2409743	C	G	0.0111	0.0014	3.0E-15	62
rs7817581	G	A	0.0086	0.0015	7.7E-09	33
rs10503555	A	G	0.0078	0.0014	3.9E-08	30
rs884152	G	T	-0.0085	0.0015	6.0E-09	34
rs7012648	G	A	-0.0097	0.0014	8.4E-12	47
rs4739558	A	G	0.0083	0.0014	6.7E-09	34
rs10503246	A	G	-0.0097	0.0015	4.6E-10	39
rs77976727	C	T	-0.0151	0.0024	3.6E-10	39
rs10095724	G	A	0.0094	0.0015	1.4E-10	41
rs10111937	C	T	-0.0084	0.0015	4.3E-08	30
rs7814267	A	G	-0.0109	0.0018	2.5E-09	36
rs7840305	A	G	0.0081	0.0014	2.7E-08	31
rs13254613	A	C	-0.0126	0.0015	1.6E-17	73
rs35918296 [a]	C	T	0.0167	0.0014	1.4E-31	137
rs11777719	A	G	-0.0117	0.0016	7.8E-14	56
rs13256357	C	T	-0.0137	0.0018	5.9E-15	61
rs7020564	A	T	0.0100	0.0016	1.1E-10	42
rs957512	T	C	0.0103	0.0015	4.5E-12	48
rs10116891	G	A	-0.0136	0.0023	5.8E-09	34
rs2275241	G	A	-0.0104	0.0014	7.5E-13	51
rs10962279	T	C	-0.0102	0.0017	1.8E-09	36
rs201666051 [a]	C	T	-0.0082	0.0014	1.3E-08	32
rs3118252	G	C	-0.0083	0.0014	6.0E-09	34
rs1935354	T	C	-0.0101	0.0014	6.1E-13	52
rs818898 [a]	A	G	0.0121	0.0015	2.3E-15	63
rs1619120	A	G	-0.0081	0.0014	1.8E-08	32
rs4744246	A	G	-0.0158	0.0015	1.6E-26	114

MR: Mendelian randomisation; LADA: latent autoimmune diabetes in adults.

[a] Corresponding proxy SNPs for rs2303384, rs116293915, rs3993347, rs200744777, rs7606059, rs636402, rs112898427, rs4688359, rs75001243, rs35918296, rs201666051, and rs818898: rs2303385, rs11832772, rs12148050, rs6107853, rs1728667, rs532425, rs4671795, rs149329399, rs13247665, rs9942789, rs7873691, and rs2821441.

ESM Table 2. Detailed information on 275 instrumental variables for MR analysis on childhood body size and subtypes of type 2 diabetes

SNP	Effect allele	Other allele	SNP-childhood body size association			
			BETA	SE	*p*	F statistic
rs1000471	C	T	-0.0095	0.0017	3.6E-08	30
rs10095724	G	A	0.0094	0.0015	1.4E-10	41
rs10111937	C	T	-0.0084	0.0015	4.3E-08	30
rs10116891	G	A	-0.0136	0.0023	5.8E-09	34
rs10133279	C	T	-0.0090	0.0014	2.8E-10	40
rs1013737	G	C	-0.0106	0.0014	3.4E-14	57
rs10182458	A	G	-0.0359	0.0014	3.5E-145	658
rs10234366	G	A	-0.0140	0.0023	1.1E-09	37
rs10498713	G	T	-0.0118	0.0020	2.0E-09	36
rs10503246	A	G	-0.0097	0.0015	4.6E-10	39
rs10503555	A	G	0.0078	0.0014	3.9E-08	30
rs1061072	G	A	0.0127	0.0023	2.4E-08	31
rs10790809	A	G	-0.0097	0.0014	7.0E-12	47
rs10791902	C	T	-0.0079	0.0014	3.6E-08	30
rs10796828	T	G	-0.0113	0.0015	6.9E-15	61
rs10823504	G	A	0.0159	0.0029	3.4E-08	30
rs10841379	A	G	-0.0084	0.0015	2.7E-08	31
rs10842356	A	T	0.0082	0.0014	4.1E-09	35
rs10860295	T	C	-0.0085	0.0014	1.6E-09	36
rs10887571	C	T	-0.0080	0.0014	1.9E-08	32
rs10896348	T	C	0.0119	0.0016	3.3E-14	58
rs10937241	A	G	-0.0109	0.0019	1.1E-08	33
rs10953577	T	C	-0.0086	0.0014	3.0E-09	35
rs10962279	T	C	-0.0102	0.0017	1.8E-09	36
rs11040333	G	A	-0.0085	0.0015	3.1E-08	31
rs11150745	A	G	0.0122	0.0015	7.5E-16	65
rs11165687	C	T	-0.0081	0.0014	1.0E-08	33
rs11205303	T	C	0.0096	0.0014	1.8E-11	45
rs11209943	A	G	-0.0183	0.0014	8.8E-38	165
rs11215403	G	A	0.0132	0.0016	5.5E-16	66
rs11256627	G	A	-0.0089	0.0015	9.5E-09	33
rs112898427 [a]	C	T	0.0103	0.0015	2.5E-11	46
rs11525873	T	C	0.0175	0.0024	1.3E-13	55
rs116293915 [a]	A	C	-0.0080	0.0014	1.5E-08	32
rs116399833	C	A	-0.0123	0.0017	5.3E-13	52
rs11642090	T	C	-0.0117	0.0015	1.0E-15	64
rs11727676	T	C	0.0131	0.0024	3.0E-08	31
rs117455294	C	A	0.0193	0.0032	1.1E-09	37
rs1177279	A	G	0.0088	0.0016	1.6E-08	32
rs11777719	A	G	-0.0117	0.0016	7.8E-14	56
rs11891707	T	C	0.0121	0.0021	3.8E-09	35
rs11925138	G	A	0.0132	0.0024	3.1E-08	31
rs1199333	G	T	0.0135	0.0018	5.9E-14	56
rs12042908	A	G	0.0275	0.0014	2.5E-84	379

SNP	Effect allele	Other allele	SNP-childhood body size association			
			BETA	SE	*p*	F statistic
rs12045879	C	T	0.0099	0.0015	5.3E-11	43
rs12110721	G	A	-0.0189	0.0019	1.5E-23	100
rs12132598	A	G	-0.0083	0.0015	2.2E-08	31
rs12140153	G	T	0.0218	0.0025	6.0E-19	79
rs12214497	G	T	0.0105	0.0015	9.4E-13	51
rs12308065	A	G	-0.0083	0.0015	1.2E-08	32
rs12429545	G	A	-0.0206	0.0021	1.2E-22	96
rs12601380	A	C	0.0078	0.0014	3.9E-08	30
rs12606230	T	C	-0.0127	0.0017	1.7E-14	59
rs12641981	C	T	-0.0222	0.0014	1.8E-55	246
rs12713889	T	C	0.0117	0.0015	3.8E-15	62
rs12748436	C	G	-0.0156	0.0026	3.5E-09	35
rs12798028	C	T	-0.0146	0.0014	1.3E-24	105
rs12817542	C	T	-0.0176	0.0029	1.8E-09	36
rs12941038	C	T	-0.0095	0.0017	1.1E-08	33
rs12992672	G	A	-0.0433	0.0019	4.0E-121	548
rs13047416	C	G	0.0123	0.0015	1.8E-17	72
rs13254613	A	C	-0.0126	0.0015	1.6E-17	73
rs13256357	C	T	-0.0137	0.0018	5.9E-15	61
rs1342831	T	C	-0.0225	0.0030	9.0E-14	56
rs1357798	C	T	-0.0094	0.0017	4.1E-08	30
rs1384660	G	A	0.0158	0.0018	1.8E-18	77
rs1402989	C	T	-0.0078	0.0014	2.7E-08	31
rs1421085	T	C	-0.0474	0.0014	5.7E-242	1103
rs1422067	C	T	0.0109	0.0016	3.8E-11	44
rs1452991	G	A	-0.0101	0.0015	5.3E-12	48
rs1476698	A	G	0.0082	0.0014	1.4E-08	32
rs1552759	T	C	0.0092	0.0015	6.2E-10	38
rs1576655	A	C	-0.0118	0.0015	4.4E-16	66
rs1619120	A	G	-0.0081	0.0014	1.8E-08	32
rs1666132	C	T	0.0083	0.0014	6.7E-09	34
rs16839832	G	T	-0.0141	0.0026	3.5E-08	30
rs16996644	C	G	-0.0193	0.0021	4.8E-20	84
rs17399739	A	G	-0.0210	0.0028	3.9E-14	57
rs17637472	G	A	-0.0109	0.0014	4.2E-14	57
rs1775255	G	T	-0.0133	0.0014	2.1E-21	90
rs1800437	G	C	0.0118	0.0018	2.6E-11	44
rs1808579	C	T	0.0092	0.0014	7.0E-11	43
rs1852006	G	A	0.0088	0.0015	1.9E-09	36
rs1865719	A	G	-0.0109	0.0015	8.5E-14	56
rs1933437	G	A	0.0142	0.0014	1.2E-22	96
rs1935354	T	C	-0.0101	0.0014	6.1E-13	52
rs200744777 [a]	T	G	-0.0121	0.0014	1.5E-17	73
rs201666051 [a]	C	T	-0.0082	0.0014	1.3E-08	32
rs2034963	G	C	0.0111	0.0015	6.7E-14	56
rs2115885	G	A	0.0106	0.0017	9.2E-10	37

SNP	Effect allele	Other allele	SNP-childhood body size association			
			BETA	SE	*p*	F statistic
rs212517	T	A	0.0088	0.0014	9.6E-10	37
rs2141004	A	C	0.0100	0.0016	2.5E-10	40
rs2175171	G	C	-0.0078	0.0014	3.6E-08	30
rs2187642	A	C	-0.0112	0.0014	1.0E-14	60
rs2207894	C	T	0.0144	0.0018	7.7E-16	65
rs2229330	T	G	-0.0197	0.0027	2.3E-13	54
rs2238435	C	G	-0.0165	0.0014	3.0E-30	131
rs2242258	T	C	-0.0092	0.0016	8.5E-09	33
rs2246623	C	T	0.0089	0.0014	2.3E-10	40
rs2268762	A	G	-0.0080	0.0014	2.6E-08	31
rs2275241	G	A	-0.0104	0.0014	7.5E-13	51
rs2281148	T	C	-0.0098	0.0016	1.5E-09	37
rs2303384 [a]	C	T	0.0127	0.0015	2.9E-18	76
rs2307111	T	C	0.0087	0.0014	1.1E-09	37
rs2356864	G	A	-0.0086	0.0014	8.8E-10	38
rs2409743	C	G	0.0111	0.0014	3.0E-15	62
rs2539692	T	A	0.0081	0.0014	1.8E-08	32
rs2594994	T	A	0.0154	0.0018	3.1E-17	71
rs262338	G	T	-0.0088	0.0014	4.7E-10	39
rs2629881	C	T	-0.0106	0.0017	3.8E-10	39
rs2722406	C	T	-0.0122	0.0016	4.4E-15	61
rs2735556	T	C	0.0172	0.0022	5.1E-15	61
rs2755253	C	T	0.0087	0.0015	1.5E-08	32
rs2767486	A	G	-0.0154	0.0017	1.2E-18	78
rs28629903	T	C	0.0091	0.0014	1.3E-10	41
rs2867116	C	A	-0.0123	0.0020	1.4E-09	37
rs2902142	C	T	0.0087	0.0014	1.7E-09	36
rs2939931	T	C	-0.0079	0.0014	2.2E-08	31
rs2958542	C	T	0.0082	0.0015	1.8E-08	32
rs2968973	C	T	0.0099	0.0015	1.1E-11	46
rs2970356	C	G	-0.0105	0.0016	4.1E-11	44
rs3013431	C	T	0.0087	0.0014	1.3E-09	37
rs3118252	G	C	-0.0083	0.0014	6.0E-09	34
rs3129942	G	T	-0.0139	0.0016	2.7E-18	76
rs3131336	C	T	-0.0191	0.0022	2.0E-18	77
rs3181269	C	T	0.0093	0.0016	6.4E-09	34
rs34196306	G	C	-0.0200	0.0023	2.3E-18	76
rs34260097	T	G	-0.0178	0.0017	2.5E-26	113
rs34517439	C	A	-0.0138	0.0022	2.0E-10	40
rs34722008	G	A	0.0083	0.0015	1.8E-08	32
rs34811474	G	A	0.0102	0.0017	8.2E-10	38
rs35162296	C	T	-0.0201	0.0023	1.0E-18	78
rs35189091	A	G	0.0090	0.0015	5.7E-10	38
rs355748	G	T	-0.0099	0.0014	6.8E-12	47
rs35588936	C	T	0.0181	0.0029	2.9E-10	40
rs35918296 [a]	C	T	0.0167	0.0014	1.4E-31	137

SNP	Effect allele	Other allele	SNP-childhood body size association			
			BETA	SE	*p*	F statistic
rs35926495	C	T	-0.0091	0.0015	4.3E-10	39
rs3774604	C	T	0.0079	0.0015	4.9E-08	30
rs3791478	T	C	0.0125	0.0023	3.3E-08	31
rs3798519	A	C	-0.0250	0.0018	8.3E-43	188
rs3810291	G	A	-0.0147	0.0015	6.4E-23	97
rs3810304	A	G	0.0103	0.0017	8.8E-10	38
rs3815156	A	G	-0.0103	0.0018	2.3E-08	31
rs3817428	C	G	-0.0108	0.0016	1.3E-11	46
rs3936511	A	G	0.0108	0.0018	1.1E-09	37
rs39862	T	C	0.0116	0.0016	7.5E-14	56
rs3993347 [a]	T	C	0.0086	0.0015	4.4E-09	34
rs4074404	T	A	-0.0127	0.0019	4.3E-11	43
rs41310284	C	A	0.0206	0.0023	1.4E-18	77
rs4267058	T	C	0.0088	0.0014	1.2E-09	37
rs435775	A	G	-0.0101	0.0016	4.9E-10	39
rs4432271	C	T	-0.0175	0.0021	9.0E-17	69
rs4545941	T	C	-0.0109	0.0019	1.1E-08	33
rs4572029	A	G	0.0111	0.0017	2.2E-10	40
rs4677156	A	T	0.0092	0.0017	3.7E-08	30
rs4688359 [a]	C	T	0.0130	0.0014	2.1E-19	81
rs4723263	G	C	-0.0083	0.0014	3.7E-09	35
rs4739558	A	G	0.0083	0.0014	6.7E-09	34
rs4744246	A	G	-0.0158	0.0015	1.6E-26	114
rs4783789	T	C	0.0096	0.0017	1.2E-08	32
rs4808961	C	G	0.0089	0.0015	1.1E-09	37
rs4889630	T	C	0.0124	0.0018	1.7E-12	50
rs4958568	G	A	0.0100	0.0016	2.1E-10	40
rs4985555	A	G	0.0087	0.0014	4.4E-10	39
rs538579	G	C	-0.0084	0.0015	2.4E-08	31
rs543874	A	G	-0.0471	0.0017	6.0E-163	740
rs55726687	G	A	-0.0145	0.0017	3.2E-17	71
rs55880046	T	G	0.0299	0.0020	3.9E-50	222
rs56133711	G	A	-0.0162	0.0016	3.1E-24	103
rs594585	T	G	0.0080	0.0014	3.4E-08	30
rs59714050	T	A	-0.0228	0.0028	4.9E-16	66
rs6001872	A	G	0.0125	0.0015	1.7E-17	72
rs601338	G	A	0.0094	0.0014	2.1E-11	45
rs61936936	A	T	-0.0136	0.0023	6.2E-09	34
rs61937656	G	A	0.0117	0.0017	2.8E-12	49
rs62032001 [a]	A	C	-0.0104	0.0018	4.2E-09	35
rs62037365	C	G	-0.0137	0.0014	1.4E-21	91
rs62048187	G	C	-0.0084	0.0015	4.4E-08	30
rs62134189	A	G	0.0142	0.0023	1.0E-09	37
rs62425398	C	A	-0.0154	0.0023	1.5E-11	46
rs630602	G	C	-0.0101	0.0014	1.8E-12	50
rs75001243 [a]	T	C	0.0083	0.0014	5.9E-09	34

SNP	Effect allele	Other allele	SNP-childhood body size association			
			BETA	SE	*p*	F statistic
rs6449532	C	T	0.0111	0.0015	3.3E-14	58
rs6577497	A	T	0.0081	0.0014	1.8E-08	32
rs661878	A	G	0.0138	0.0021	2.5E-11	45
rs663129	G	A	-0.0333	0.0017	6.8E-90	404
rs6719507	G	A	0.0078	0.0014	2.9E-08	31
rs67603370	G	A	-0.0158	0.0027	3.7E-09	35
rs67679818	C	T	0.0078	0.0014	4.9E-08	30
rs6783281	A	G	-0.0096	0.0015	5.5E-10	38
rs68015088	G	A	0.0081	0.0015	4.3E-08	30
rs6931604	C	T	-0.0083	0.0014	6.2E-09	34
rs6974282	C	T	0.0101	0.0018	1.6E-08	32
rs6979832	A	G	-0.0094	0.0014	2.3E-11	45
rs7012648	G	A	-0.0097	0.0014	8.4E-12	47
rs7020564	A	T	0.0100	0.0016	1.1E-10	42
rs7084503	T	C	0.0129	0.0014	5.2E-20	84
rs71231793 [a]	T	C	-0.0154	0.0020	5.0E-14	57
rs7123283	C	T	0.0083	0.0014	3.7E-09	35
rs7132908	G	A	-0.0313	0.0014	1.6E-104	471
rs7145052	C	T	-0.0083	0.0014	3.7E-09	35
rs71483681 [a]	T	C	-0.0081	0.0014	1.4E-08	32
rs7159126	T	C	0.0087	0.0016	2.4E-08	31
rs7161424	G	A	-0.0102	0.0014	5.2E-13	52
rs7162542	C	G	0.0093	0.0014	4.0E-11	44
rs7163692	C	G	0.0083	0.0015	1.5E-08	32
rs7217460	G	A	0.0094	0.0017	2.4E-08	31
rs7237444	G	A	0.0101	0.0015	3.2E-11	44
rs7239114	G	A	-0.0135	0.0014	1.7E-21	91
rs72755233	G	A	-0.0176	0.0022	2.3E-15	63
rs72819571	G	T	0.0119	0.0015	7.3E-16	65
rs7305424	A	T	-0.0104	0.0015	2.4E-12	49
rs7306710	T	C	0.0100	0.0014	1.6E-12	50
rs73085586	G	A	-0.0097	0.0018	3.0E-08	31
rs73422097	A	G	-0.0106	0.0015	3.3E-12	49
rs7354849	A	G	-0.0082	0.0014	6.8E-09	34
rs7355953	T	C	-0.0152	0.0017	7.4E-19	79
rs7377083	C	A	-0.0137	0.0014	5.3E-22	93
rs7424771	G	A	0.0089	0.0014	2.2E-10	40
rs7439324	C	T	0.0108	0.0019	1.5E-08	32
rs75001243 [a]	C	T	-0.0137	0.0015	5.1E-21	89
rs7503580	C	T	-0.0111	0.0019	8.9E-09	33
rs7522014	A	G	0.0093	0.0015	9.1E-10	38
rs7536458	T	G	-0.0101	0.0016	1.9E-10	41
rs754635	C	G	-0.0159	0.0022	5.6E-13	52
rs7565437	T	C	0.0082	0.0014	9.5E-09	33
rs7606059 [a]	T	C	-0.0105	0.0015	2.1E-12	49
rs76187039	G	T	-0.0113	0.0021	3.5E-08	30

SNP	Effect allele	Other allele	SNP-childhood body size association			
			BETA	SE	*p*	F statistic
rs7619139	T	A	-0.0098	0.0014	6.5E-12	47
rs7625768	G	A	-0.0102	0.0015	1.5E-11	46
rs7633995	A	G	-0.0145	0.0025	4.9E-09	34
rs7656673	A	G	-0.0116	0.0014	6.2E-16	65
rs7672	C	G	0.0090	0.0016	7.9E-09	33
rs7711823	A	G	0.0084	0.0015	9.3E-09	33
rs7719067	A	G	0.0135	0.0014	1.8E-21	91
rs7753558	C	A	0.0091	0.0015	5.2E-10	39
rs7759938	C	T	-0.0100	0.0015	2.9E-11	44
rs77960	G	A	0.0105	0.0015	1.7E-12	50
rs77976727	C	T	-0.0151	0.0024	3.6E-10	39
rs7808296	C	T	-0.0100	0.0015	2.8E-11	44
rs7814267	A	G	-0.0109	0.0018	2.5E-09	36
rs7817581	G	A	0.0086	0.0015	7.7E-09	33
rs7840305	A	G	0.0081	0.0014	2.7E-08	31
rs78420139	G	A	0.0170	0.0031	3.5E-08	30
rs78607331	C	T	-0.0239	0.0034	1.7E-12	50
rs788858	A	G	0.0123	0.0015	1.7E-15	63
rs78907487	A	C	-0.0124	0.0020	3.9E-10	39
rs7951870	T	C	-0.0116	0.0019	5.4E-10	39
rs7958241	A	G	-0.0131	0.0015	8.6E-19	78
rs796915	C	G	-0.0129	0.0015	2.3E-17	72
rs7989098	T	C	-0.0121	0.0016	1.0E-13	55
rs8030456	C	T	0.0213	0.0017	2.8E-37	163
rs8096658	C	G	0.0090	0.0014	2.0E-10	40
rs8117463	G	A	0.0089	0.0015	2.9E-09	35
rs8130408	A	C	-0.0090	0.0016	2.3E-08	31
rs818898 [a]	A	G	0.0121	0.0015	2.3E-15	63
rs824207	A	G	-0.0093	0.0014	3.3E-11	44
rs836179	A	G	0.0098	0.0015	1.4E-11	46
rs884152	G	T	-0.0085	0.0015	6.0E-09	34
rs918472	G	A	-0.0099	0.0016	2.4E-10	40
rs9260164 [a]	C	T	-0.0152	0.0016	5.7E-21	88
rs9265968 [a]	A	T	-0.0201	0.0021	1.4E-21	91
rs9291816	C	T	0.0134	0.0015	4.2E-19	80
rs9299	C	T	-0.0096	0.0015	7.7E-11	42
rs9370527	G	A	-0.0095	0.0016	6.4E-09	34
rs9438393	A	G	0.0103	0.0014	3.4E-13	53
rs947088	G	T	-0.0106	0.0016	1.0E-11	46
rs9538146	A	G	-0.0116	0.0014	2.0E-16	68
rs957512	T	C	0.0103	0.0015	4.5E-12	48
rs9594686	C	T	0.0107	0.0018	6.4E-09	34
rs9603697	C	T	-0.0130	0.0015	4.6E-18	75
rs9610387	G	A	0.0138	0.0025	3.7E-08	30
rs9611560	T	C	0.0093	0.0016	7.6E-09	33
rs9652090	G	T	-0.0083	0.0014	4.0E-09	35

SNP	Effect allele	Other allele	SNP-childhood body size association			
			**BETA**	**SE**	***p***	**F statistic**
rs9922288	A	G	0.0105	0.0017	3.0E-10	40

MR: Mendelian randomisation.

[a] Corresponding proxy SNPs for rs112898427, rs116293915, rs200744777, rs201666051, rs2303384, rs35918296, rs3993347, rs4688359, rs62032001, rs636402, rs71231793, rs71483681, rs75001243, rs7606059, rs818898, rs9260164, and rs9265968: rs1842958, rs11832772, rs6038566, rs4977811, rs2303385, rs9942789, rs12147246, rs11130822, rs2967274, rs2577761, rs56076430, rs67582926, rs13247665, rs1706252, rs2821441, rs9260080, and rs1625792.

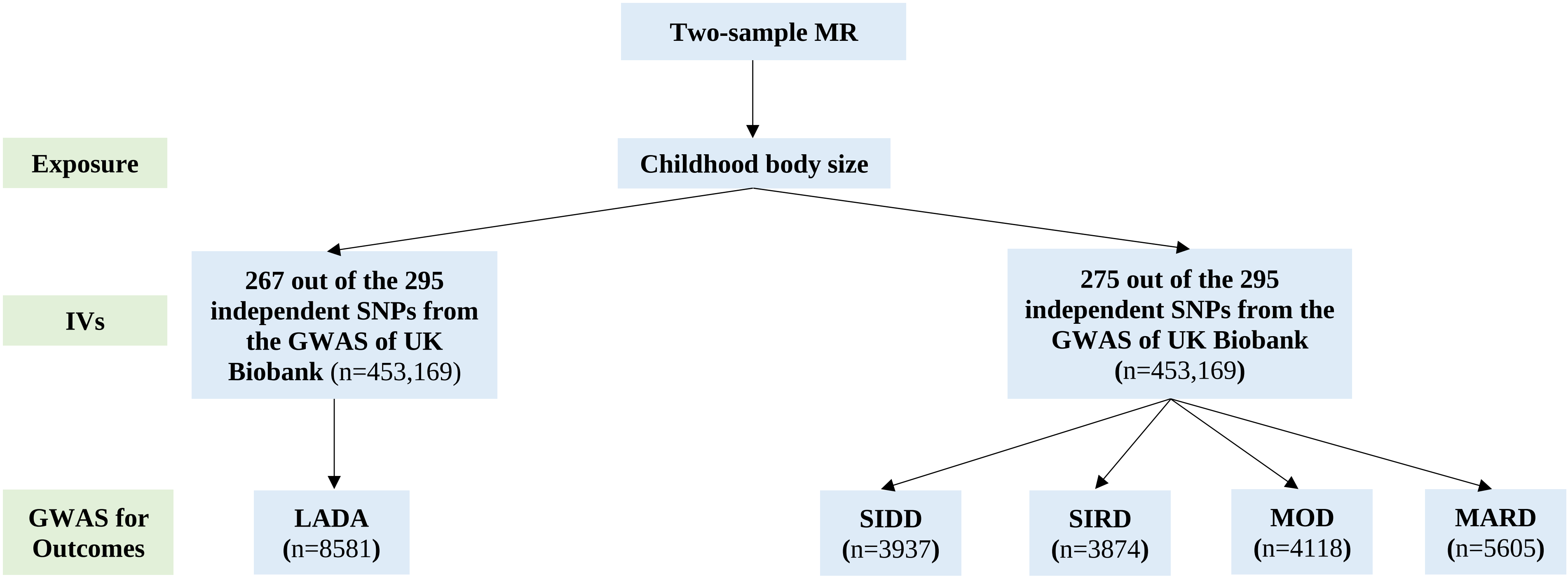

ESM Fig. 1 Flow chart of IVs used in the MR study

IV: instrumental variable; MR: Mendelian randomisation; GWAS: genome-wide association study; LADA: latent autoimmune diabetes in adults; SIDD: severe insulin-deficient diabetes; SIRD: severe insulin-resistant diabetes; MOD: mild obesity-related diabetes; MARD: mild age-related diabetes

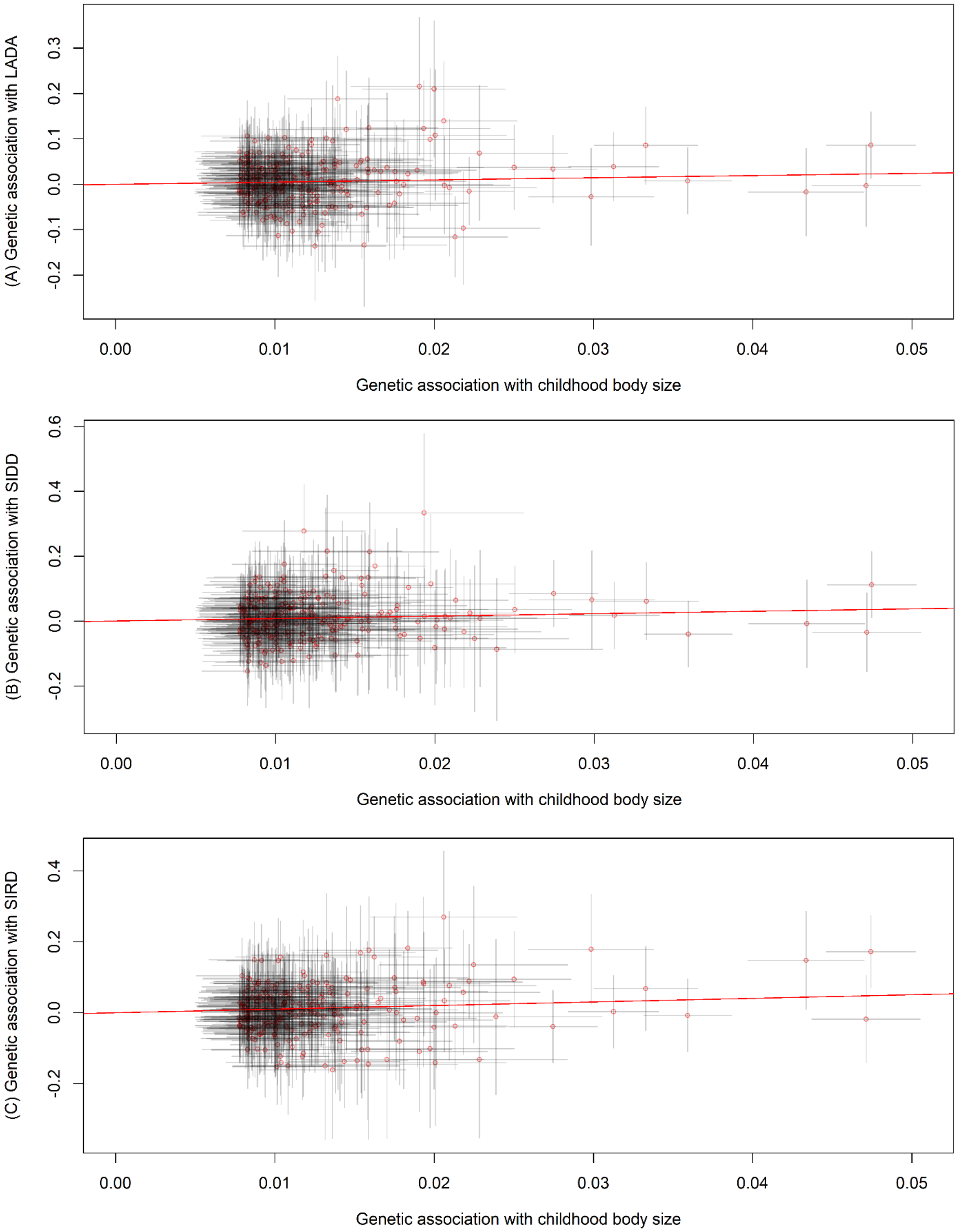
(A) Genetic association with LADA
0.3
0.2
0.1
0.0
-0.1
-0.2
0.00
0.01
0.02
0.03
0.04
0.05
Genetic association with childhood body size
(B) Genetic association with SIDD
0.6
0.4
0.2
0.0
-0.2
0.00
0.01
0.02
0.03
0.04
0.05
Genetic association with childhood body size
(C) Genetic association with SIRD
0.4
0.2
0.0
-0.2
0.00
0.01
0.02
0.03
0.04
0.05
Genetic association with childhood body size

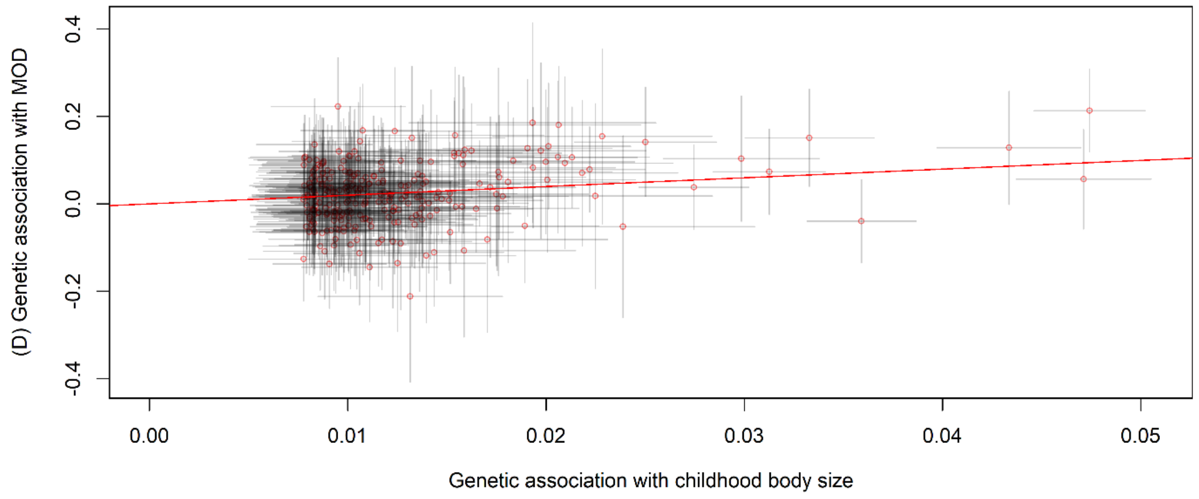

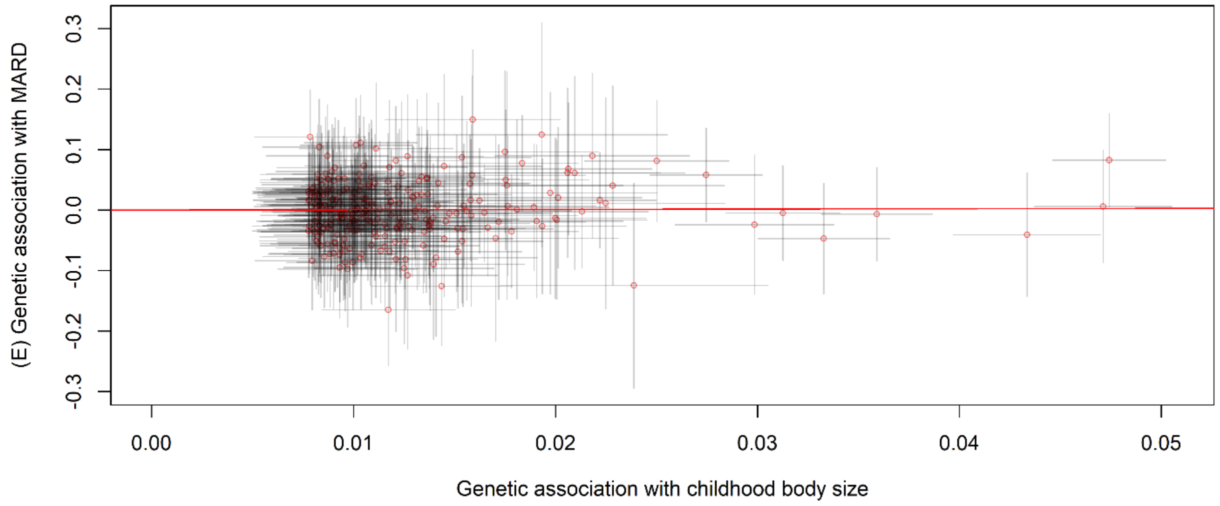

ESM Fig. 2 Scatter plots for associations of instrumental variables with childhood body size and different subtypes of adult-onset diabetes

LADA: latent autoimmune diabetes in adults; SIDD: severe insulin-deficient diabetes; SIRD: severe insulin-resistant diabetes; MOD: mild obesity-related diabetes; MARD: mild age-related diabetes. The slope of the red line is the log-odds ratio of increase in LADA risk one category increase in childhood body size (thinner, about average, or plumper), based on the inverse-variance weighted method.

Methods	No. of SNPs	OR (95% CI)	*p* for risk estimate	*p* for heterogeneity
LADA				
Robust IVW	267	1.60 (1.01, 2.52)	0.044	0.022
Weighted median	267	1.42 (0.72, 2.82)	0.312	
MR-Egger	267	2.30 (0.82, 6.44)	0.115	0.004
MR-PRESSO raw estimate	267	1.62 (1.05, 2.52)	0.032	
SIDD				
Robust IVW	275	1.77 (0.95, 3.30)	0.071	0.488
Weighted median	275	1.76 (0.70, 4.41)	0.229	
MR-Egger	275	3.51 (0.89, 13.84)	0.073	0.026
MR-PRESSO outlier-corrected estimate	274	2.02 (1.14, 3.59)	0.017	
SIRD				
Robust IVW	275	2.37 (1.17, 4.79)	0.016	0.287
Weighted median	275	2.52 (0.98, 6.47)	0.055	
MR-Egger	275	7.78 (2.05, 29.47)	0.003	0.134
MR-PRESSO raw estimate	275	2.76 (1.56, 4.88)	0.001	
MOD				
Robust IVW	275	7.39 (3.83, 14.25)	<0.001	0.003
Weighted median	275	10.30 (4.18, 25.36)	<0.001	
MR-Egger	275	21.68 (5.89, 79.75)	<0.001	0.016
MR-PRESSO outlier-corrected estimate	274	7.01 (4.04, 12.15)	<0.001	
MARD				
Robust IVW	275	1.02 (0.64, 1.60)	0.946	0.699
Weighted median	275	0.91 (0.45, 1.85)	0.793	
MR-Egger	275	1.75 (0.66, 4.64)	0.257	0.407
MR-PRESSO raw estimate	275	1.06 (0.70, 1.60)	0.785	

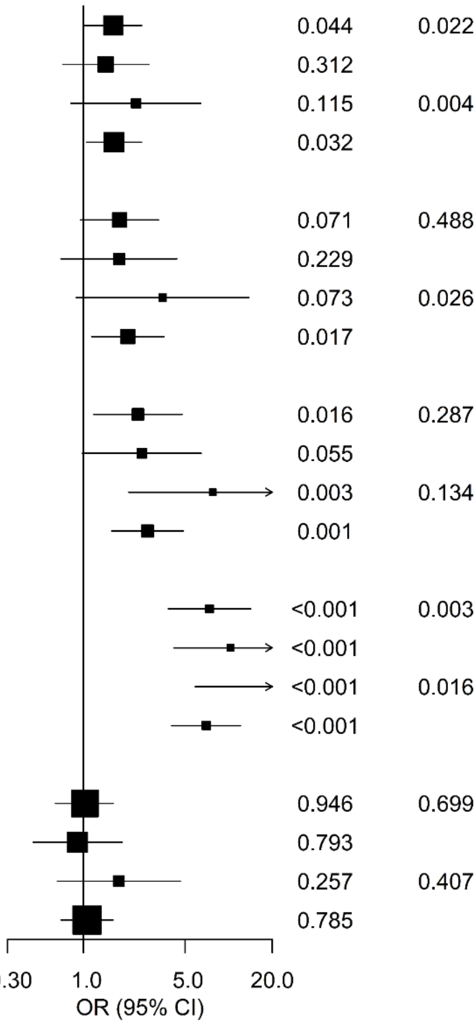

ESM Fig. 3 Genetically predicted childhood body size (thinner, about average, or plumper) and risks of diabetes subtypes based on other MR estimators

MR: Mendelian randomisation; LADA: latent autoimmune diabetes in adults; SIDD: severe insulin-deficient diabetes; SIRD: severe insulin-resistant diabetes; MOD: mild obesity-related diabetes; MARD: mild age-related diabetes; IVW: inverse-variance weighted; MR-Egger: Egger regression of MR; MR-PRESSO: MR pleiotropy residual sum and outlier approach.

MR-Egger intercept: -0.005, p for directional pleiotropy: 0.467 in the analysis of LADA; intercept: -0.007, p for directional pleiotropy: 0.423 in the analysis of SIDD; intercept: -0.015, p for directional pleiotropy: 0.092 in the analysis of SIRD; intercept: -0.016; p for directional pleiotropy: 0.070 in the analysis of MOD; intercept: −0.007, p for directional pleiotropy: 0.261 in the analysis of MARD.

MR-PRESSO detected rs10498713 as the outlier for the analysis of SIDD (*p* for global test of pleiotropy: 0.03) and the outlier-corrected estimate was presented (*p* for test of distortion in estimate by the outlier: 0.872). MR-PRESSO detected rs1000471 as the outlier for the analysis of MOD (*p* for global test of pleiotropy: 0.013; *p* for test of distortion in estimate by the outlier: 0.894). No outlier was detected in the analysis of other subtypes of adult-onset diabetes.

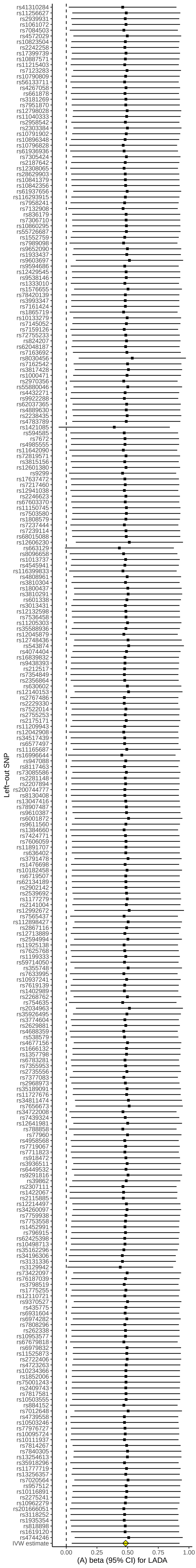
Left-out SNP
IVW estimate
0.00
0.25
0.50
0.75
1.00
(A) beta (95% CI) for LADA

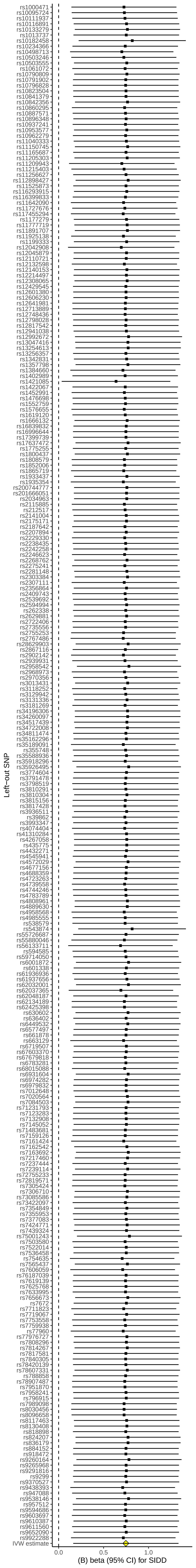

Left−out SNP
rs1000471
rs10095724
rs10111937
rs10116891
rs10133279
rs1013737
rs10182458
rs10234366
rs10498713
rs10503246
rs10503555
rs1061072
rs10790809
rs10791902
rs10796828
rs10823504
rs10841379
rs10842356
rs10860295
rs10887571
rs10896348
rs10937241
rs10953577
rs10962279
rs11040333
rs11150745
rs11165687
rs11205303
rs11209943
rs11215403
rs11256627
rs112898427
rs11525873
rs116293915
rs116399833
rs11642090
rs11727676
rs117455294
rs1177279
rs11777719
rs11891707
rs11925138
rs1199333
rs12042908
rs12045879
rs12110721
rs12132598
rs12140153
rs12214497
rs12308065
rs12429545
rs12601380
rs12606230
rs12641981
rs12713889
rs12748436
rs12798028
rs12817542
rs12941038
rs12992672
rs13047416
rs13254613
rs13256357
rs1342831
rs1357798
rs1384660
rs1402989
rs1421085
rs1422067
rs1452991
rs1476698
rs1552759
rs1576655
rs1619120
rs1666132
rs16839832
rs16996644
rs17399739
rs17637472
rs1775255
rs1800437
rs1808579
rs1852006
rs1865719
rs1933437
rs1935354
rs200744777
rs201666051
rs2034963
rs2115885
rs212517
rs2141004
rs2175171
rs2187642
rs2207894
rs2229330
rs2238435
rs2242258
rs2246623
rs2268762
rs2275241
rs2281148
rs2303384
rs2307111
rs2356864
rs2409743
rs2539692
rs2594994
rs262338
rs2629881
rs2722406
rs2735556
rs2755253
rs2767486
rs28629903
rs2867116
rs2902142
rs2939931
rs2958542
rs2968973
rs2970356
rs3013431
rs3118252
rs3129942
rs3131336
rs3181269
rs34196306
rs34260097
rs34517439
rs34722008
rs34811474
rs35162296
rs35189091
rs355748
rs35588936
rs35918296
rs35926495
rs3774604
rs3791478
rs3798519
rs3810291
rs3810304
rs3815156
rs3817428
rs3936511
rs39862
rs3993347
rs4074404
rs41310284
rs4267058
rs435775
rs4432271
rs4545941
rs4572029
rs4677156
rs4688359
rs4723263
rs4739558
rs4744246
rs4783789
rs4808961
rs4889630
rs4958568
rs4985555
rs538579
rs543874
rs55726687
rs55880046
rs56133711
rs594585
rs59714050
rs6001872
rs61936936
rs61937656
rs62032001
rs62037365
rs62048187
rs62134189
rs62425398
rs630602
rs636402
rs6449532
rs6577497
rs661878
rs663129
rs6719507
rs67603370
rs67679818
rs6783281
rs68015088
rs6931604
rs6974282
rs6979832
rs7012648
rs7020564
rs7084503
rs71231793
rs7123283
rs7132908
rs7145052
rs71483681
rs7159126
rs7161424
rs7162542
rs7163692
rs7217460
rs7237444
rs7239114
rs72755233
rs72819571
rs7305424
rs7306710
rs73085586
rs73422097
rs7354849
rs7355953
rs7377083
rs7424771
rs7439324
rs75001243
rs7503580
rs7522014
rs7536458
rs754635
rs7565437
rs7606059
rs76187039
rs7619139
rs7625768
rs7633995
rs7656673
rs7672
rs7711823
rs7719067
rs7753558
rs7759938
rs77960
rs77976727
rs7808296
rs7814267
rs7817581
rs7840305
rs78420139
rs78607331
rs788858
rs78907487
rs7951870
rs7958241
rs796915
rs7989098
rs8030456
rs8096658
rs8117463
rs8130408
rs818898
rs824207
rs836179
rs884152
rs918472
rs9260164
rs9265968
rs9291816
rs9299
rs9370527
rs9438393
rs947088
rs9538146
rs957512
rs9594686
rs9603697
rs9610387
rs9611560
rs9652090
rs9922288
IVW estimate
0.0
0.5
1.0
(B) beta (95% CI) for SIDD

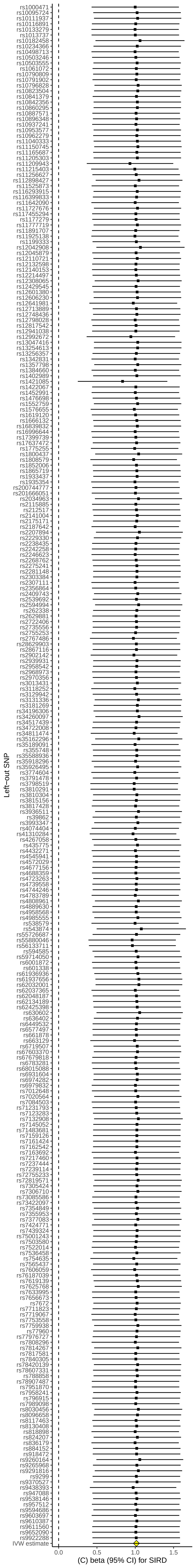

Left-out SNP
rs1000471
rs10095724
rs10111937
rs10116891
rs10133279
rs1013737
rs10182458
rs10234366
rs10498713
rs10503246
rs10503555
rs1061072
rs10790809
rs10791902
rs10796828
rs10823504
rs10841379
rs10842356
rs10860295
rs10887571
rs10896348
rs10937241
rs10953577
rs10962279
rs11040333
rs11150745
rs11165687
rs11205303
rs11209943
rs11215403
rs11256627
rs112898427
rs11525873
rs116293915
rs116399833
rs11642090
rs11727676
rs117455294
rs1177279
rs11777719
rs11891707
rs11925138
rs1199333
rs12042908
rs12045879
rs12110721
rs12132598
rs12140153
rs12214497
rs12308065
rs12429545
rs12601380
rs12606230
rs12641981
rs12713889
rs12748436
rs12798028
rs12817542
rs12941038
rs12992672
rs13047416
rs13254613
rs13256357
rs1342831
rs1357798
rs1384660
rs1402989
rs1421085
rs1422067
rs1452991
rs1476698
rs1552759
rs1576655
rs1619120
rs1666132
rs16839832
rs16996644
rs17399739
rs17637472
rs1775255
rs1800437
rs1808579
rs1852006
rs1865719
rs1933437
rs1935354
rs200744777
rs201666051
rs2034963
rs2115885
rs212517
rs2141004
rs2175171
rs2187642
rs2207894
rs2229330
rs2238435
rs2242258
rs2246623
rs2268762
rs2275241
rs2281148
rs2303384
rs2307111
rs2356864
rs2409743
rs2539692
rs2594994
rs262338
rs2629881
rs2722406
rs2735556
rs2755253
rs2767486
rs28629903
rs2867116
rs2902142
rs2939931
rs2958542
rs2968973
rs2970356
rs3013431
rs3118252
rs3129942
rs3131336
rs3181269
rs34196306
rs34260097
rs34517439
rs34722008
rs34811474
rs35162296
rs35189091
rs355748
rs35588936
rs35918296
rs35926495
rs3774604
rs3791478
rs3798519
rs3810291
rs3810304
rs3815156
rs3817428
rs3936511
rs39862
rs3993347
rs4074404
rs41310284
rs4267058
rs435775
rs4432271
rs4545941
rs4572029
rs4677156
rs4688359
rs4723263
rs4739558
rs4744246
rs4783789
rs4808961
rs4889630
rs4958568
rs4985555
rs538579
rs543874
rs55726687
rs55880046
rs56133711
rs594585
rs59714050
rs6001872
rs601338
rs61936936
rs61937656
rs62032001
rs62037365
rs62048187
rs62134189
rs62425398
rs630602
rs636402
rs6449532
rs6577497
rs661878
rs663129
rs6719507
rs67603370
rs67679818
rs6783281
rs68015088
rs6931604
rs6974282
rs6979832
rs7012648
rs7020564
rs7084503
rs71231793
rs7123283
rs7132908
rs7145052
rs71483681
rs7159126
rs7161424
rs7162542
rs7163692
rs7217460
rs7237444
rs7239114
rs72755233
rs72819571
rs7305424
rs7306710
rs73085586
rs73422097
rs7354849
rs7355953
rs7377083
rs7424771
rs7439324
rs75001243
rs7503580
rs7522014
rs7536458
rs754635
rs7565437
rs7606059
rs76187039
rs7619139
rs7625768
rs7633995
rs7656673
rs7672
rs7711823
rs7719067
rs7753558
rs7759938
rs77960
rs77976727
rs7808296
rs7814267
rs7817581
rs7840305
rs78420139
rs78607331
rs788858
rs78907487
rs7951870
rs7958241
rs796915
rs7989098
rs8030456
rs8096658
rs8117463
rs8130408
rs818898
rs824207
rs836179
rs884152
rs918472
rs9260164
rs9265968
rs9291816
rs9299
rs9370527
rs9438393
rs947088
rs9538146
rs957512
rs9594686
rs9603697
rs9610387
rs9611560
rs9652090
rs9922288
IVW estimate
0.0
0.5
1.0
1.5
(C) beta (95% CI) for SIRD

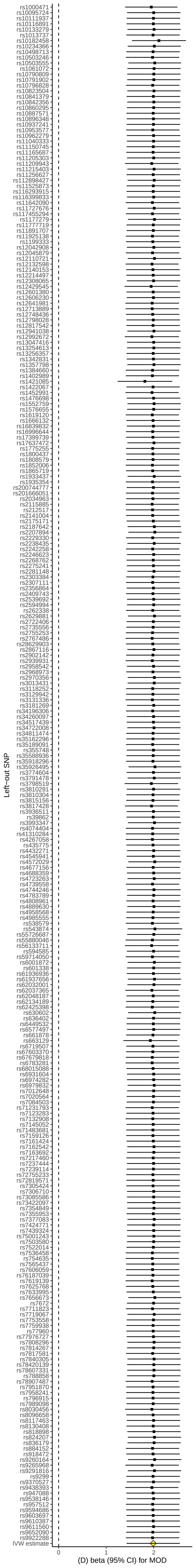
rs1000471
rs10095724
rs10111937
rs10116891
rs10133279
rs1013737
rs10182458
rs10234366
rs10498713
rs10503246
rs10503555
rs1061072
rs10790809
rs10791902
rs10796828
rs10823504
rs10841379
rs10842356
rs10860295
rs10887571
rs10896348
rs10937241
rs10953577
rs10962279
rs11040333
rs11150745
rs11165687
rs11205303
rs11209943
rs11215403
rs11256627
rs112898427
rs11525873
rs116293915
rs116399833
rs11642090
rs11727676
rs117455294
rs1177279
rs11777719
rs11891707
rs11925138
rs1199333
rs12042908
rs12045879
rs12110721
rs12132598
rs12140153
rs12214497
rs12308065
rs12429545
rs12601380
rs12606230
rs12641981
rs12713889
rs12748436
rs12798028
rs12817542
rs12941038
rs12992672
rs13047416
rs13254613
rs13256357
rs1342831
rs1357798
rs1384660
rs1402989
rs1421085
rs1422067
rs1452991
rs1476698
rs1552759
rs1576655
rs1619120
rs1666132
rs16839832
rs16996644
rs17399739
rs17637472
rs1775255
rs1800437
rs1808579
rs1852006
rs1865719
rs1933437
rs1935354
rs200744777
rs201666051
rs2034963
rs2115885
rs212517
rs2141004
rs2175171
rs2187642
rs2207894
rs2229330
rs2238435
rs2242258
rs2246623
rs2268762
rs2275241
rs2281148
rs2303384
rs2307111
rs2356864
rs2409743
rs2539692
rs2594994
rs262338
rs2629881
rs2722406
rs2735556
rs2755253
rs2767486
rs28629903
rs2867116
rs2902142
rs2939931
rs2958542
rs2968973
rs2970356
rs3013431
rs3118252
rs3129942
rs3131336
rs3181269
rs34196306
rs34260097
rs34517439
rs34722008
rs34811474
rs35162296
rs35189091
rs355748
rs35588936
rs35918296
rs35926495
rs3774604
rs3791478
rs3798519
rs3810291
rs3810304
rs3815156
rs3817428
rs3936511
rs39862
rs3993347
rs4074404
rs41310284
rs4267058
rs435775
rs4432271
rs4545941
rs4572029
rs4677156
rs4688359
rs4723263
rs4739558
rs4744246
rs4783789
rs4808961
rs4889630
rs4958568
rs4985555
rs538579
rs543874
rs55726687
rs55880046
rs56133711
rs594585
rs59714050
rs6001872
rs601338
rs61936936
rs61937656
rs62032001
rs62037365
rs62048187
rs62134189
rs62425398
rs630602
rs636402
rs6449532
rs6577497
rs661878
rs663129
rs6719507
rs67603370
rs67679818
rs6783281
rs68015088
rs6931604
rs6974282
rs6979832
rs7012648
rs7020564
rs7084503
rs71231793
rs7123283
rs7132908
rs7145052
rs71483681
rs7159126
rs7161424
rs7162542
rs7163692
rs7217460
rs7237444
rs7239114
rs72755233
rs72819571
rs7305424
rs7306710
rs73085586
rs73422097
rs7354849
rs7355953
rs7377083
rs7424771
rs7439324
rs75001243
rs7503580
rs7522014
rs7536458
rs754635
rs7565437
rs7606059
rs76187039
rs7619139
rs7625768
rs7633995
rs7656673
rs7672
rs7711823
rs7719067
rs7753558
rs7759938
rs77960
rs77976727
rs7808296
rs7814267
rs7817581
rs7840305
rs78420139
rs78607331
rs788858
rs78907487
rs7951870
rs7958241
rs796915
rs7989098
rs8030456
rs8096658
rs8117463
rs8130408
rs818898
rs824207
rs836179
rs884152
rs918472
rs9260164
rs9265968
rs9291816
rs9299
rs9370527
rs9438393
rs947088
rs9538146
rs957512
rs9594686
rs9603697
rs9610387
rs9611560
rs9652090
rs9922288
IVW estimate
Left−out SNP
0
1
2
(D) beta (95% CI) for MOD

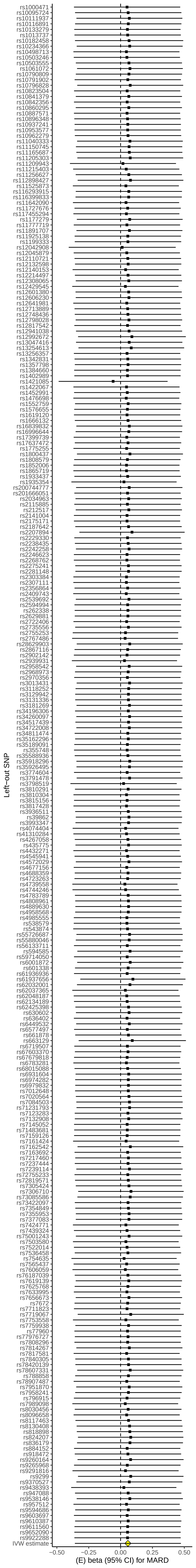
rs1000471
rs10095724
rs10111937
rs10116891
rs10133279
rs1013737
rs10182458
rs10234366
rs10498713
rs10503246
rs10503555
rs1061072
rs10790809
rs10791902
rs10796828
rs10823504
rs10841379
rs10842356
rs10860295
rs10887571
rs10896348
rs10937241
rs10953577
rs10962279
rs11040333
rs11150745
rs11165687
rs11205303
rs11209943
rs11215403
rs11256627
rs112898427
rs11525873
rs116293915
rs116399833
rs11642090
rs11727676
rs117455294
rs1177279
rs11777719
rs11891707
rs11925138
rs1199333
rs12042908
rs12045879
rs12110721
rs12132598
rs12140153
rs12214497
rs12308065
rs12429545
rs12601380
rs12606230
rs12641981
rs12713889
rs12748436
rs12798028
rs12817542
rs12941038
rs12992672
rs13047416
rs13254613
rs13256357
rs1342831
rs1357798
rs1384660
rs1402989
rs1421085
rs1422067
rs1452991
rs1476698
rs1552759
rs1576655
rs1619120
rs1666132
rs16839832
rs16996644
rs17399739
rs17637472
rs1775255
rs1800437
rs1808579
rs1852006
rs1865719
rs1933437
rs1935354
rs200744777
rs201666051
rs2034963
rs2115885
rs212517
rs2141004
rs2175171
rs2187642
rs2207894
rs2229330
rs2238435
rs2242258
rs2246623
rs2268762
rs2275241
rs2281148
rs2303384
rs2307111
rs2356864
rs2409743
rs2539692
rs2594994
rs262338
rs2629881
rs2722406
rs2735556
rs2755253
rs2767486
rs28629903
rs2867116
rs2902142
rs2939931
rs2958542
rs2968973
rs2970356
rs3013431
rs3118252
rs3129942
rs3131336
rs3181269
rs34196306
rs34260097
rs34517439
rs34722008
rs34811474
rs35162296
rs35189091
rs355748
rs35588936
rs35918296
rs35926495
rs3774604
rs3791478
rs3798519
rs3810291
rs3810304
rs3815156
rs3817428
rs3936511
rs39862
rs3993347
rs4074404
rs41310284
rs4267058
rs435775
rs4432271
rs4545941
rs4572029
rs4677156
rs4688359
rs4723263
rs4739558
rs4744246
rs4783789
rs4808961
rs4889630
rs4958568
rs4985555
rs538579
rs543874
rs55726687
rs55880046
rs56133711
rs594585
rs59714050
rs6001872
rs601338
rs61936936
rs61937656
rs62032001
rs62037365
rs62048187
rs62134189
rs62425398
rs630602
rs636402
rs6449532
rs6577497
rs661878
rs663129
rs6719507
rs67603370
rs67679818
rs6783281
rs68015088
rs6931604
rs6974282
rs6979832
rs7012648
rs7020564
rs7084503
rs71231793
rs7123283
rs7132908
rs7145052
rs71483681
rs7159126
rs7161424
rs7162542
rs7163692
rs7217460
rs7237444
rs7239114
rs72755233
rs72819571
rs7305424
rs7306710
rs73085586
rs73422097
rs7354849
rs7355953
rs7377083
rs7424771
rs7439324
rs75001243
rs7503580
rs7522014
rs7536458
rs754635
rs7565437
rs7606059
rs76187039
rs7619139
rs7625768
rs7633995
rs7656673
rs7672
rs7711823
rs7719067
rs7753558
rs7759938
rs77960
rs77976727
rs7808296
rs7814267
rs7817581
rs7840305
rs78420139
rs78607331
rs788858
rs78907487
rs7951870
rs7958241
rs796915
rs7989098
rs8030456
rs8096658
rs8117463
rs8130408
rs818898
rs824207
rs836179
rs884152
rs918472
rs9260164
rs9265968
rs9291816
rs9299
rs9370527
rs9438393
rs947088
rs9538146
rs957512
rs9594686
rs9603697
rs9610387
rs9611560
rs9652090
rs9922288
IVW estimate
Left-out SNP
-0.50
-0.25
0.00
0.25
0.50
(E) beta (95% CI) for MARD

ESM Fig. 4 MR analysis on the associations between childhood body size and diabetes subtypes by leaving one SNP out from the IVs each time.

(A) Leave-one-out analysis for LADA; (B) Leave-one-out analysis for SIDD; (C) Leave-one-out analysis for SIRD; (D) Leave-one-out analysis for MOD; (E) Leave-one-out analysis for MARD

The estimates were presented as beta (the logarithm of ORs).

MR: Mendelian randomisation; IV: instrumental variable; LADA: latent autoimmune diabetes in adults; SIDD: severe insulin-deficient diabetes; SIRD: severe insulin-resistant diabetes; MOD: mild obesity-related diabetes; MARD: mild age-related diabetes; IVW: inverse-variance weighted.

References

1. Bulik-Sullivan B, Finucane HK, Anttila V, et al. An atlas of genetic correlations across human diseases and traits. Nat Genet 2015;47(11):1236-41. (In eng). DOI: 10.1038/ng.3406.
2. Bulik-Sullivan BK, Loh PR, Finucane HK, et al. LD Score regression distinguishes confounding from polygenicity in genome-wide association studies. Nat Genet 2015;47(3):291-5. (In eng). DOI: 10.1038/ng.3211.
